# Synergistic impact of macrolide resistance and H3N2 infection on *M. pneumoniae* outbreak in children

**DOI:** 10.1128/spectrum.01844-24

**Published:** 2025-02-25

**Authors:** Jiali Chen, Yingdan Wang, Juan Cheng, Yunping Ma, Xin Zhang, Xuezhou Bai, Palizhati Rehati, Huashun Cui, Fan Wu, Qiuhui Pan, Jinghe Huang

**Affiliations:** 1 Key Laboratory of Medical Molecular Virology (MOE/NHC/CAMS), Shanghai Institute of Infectious Disease and Biosecurity, Shanghai Frontiers Science Center of Pathogenic Microorganisms and Infection, School of Basic Medical Sciences, Fudan University, Shanghai, China; 2 Clinical Laboratory, Shanghai Children’s Medical Center, School of Medicine, Shanghai Jiao Tong University, Shanghai, China; 3 Shanghai Immune Therapy Institute, Shanghai Jiao Tong University School of Medicine Affiliated Renji Hospital 71140 , Shanghai, China; 4 Department of Acupuncture and Moxibustion, Shanghai Shuguang Hospital Affiliated to Shanghai University of Traditional Chinese Medicine 66329 , Shanghai, China; 5 Faculty of Medical Laboratory Science, College of Health Science and Technology, Shanghai Jiao Tong University School of Medicine, Shanghai, China; 6 Shanghai Key Laboratory of Clinical Molecular Diagnostics for Pediatrics, Shanghai, China; bioMerieux Inc., Denver, Colorado, USA

**Keywords:** *Mycoplasma pneumoniae *infections, H3N2 influenza, SARS-CoV-2, neutralizing Ab

## Abstract

In November 2023, there was a substantial increase in the incidence of *Mycoplasma pneumoniae* infections in China following waves of SARS-CoV-2 Omicron variant and influenza outbreaks. This study aimed to elucidate the epidemiological features and clinical implications of *M. pneumoniae* infections in children and explore the potential influence of SARS-CoV-2 Omicron variants and influenza A infections on the *M. pneumoniae* outbreak. Among 38,668 children with lower respiratory tract infections from January to December 2023, 11,919 tested positive for *M. pneumoniae*, predominantly between October and December. The majority of the children with *M. pneumoniae* were aged 5–10 years, with type 1 strains and macrolide-resistant *M. pneumoniae* strains having the highest prevalence rates. Statistical analysis revealed elevated C-reactive protein, neutrophil, and monocyte levels and decreased lymphocyte, basophil, and eosinophil counts in *M. pneumoniae*-positive children. *M. pneumoniae*-positive children also presented significantly increased neutralizing antibody levels against preceding influenza A (H3N2) but not against SARS-CoV-2 Omicron variants. A parallel trend was observed between *M. pneumoniae* and H3N2 prevalence from June to December 2023. The emergence of macrolide-resistant strains and prior influenza A (H3N2) epidemics notably contributed to the *M. pneumoniae* outbreak. These findings suggested that H3N2 infection facilitates *M. pneumoniae* infection through various mechanisms. This study underscores the complex interactions between respiratory pathogens and highlights the need for comprehensive surveillance and response strategies.

**IMPORTANCE:**

This study identified key factors contributing to an outbreak of *Mycoplasma pneumoniae* that affected 11,919 children. The influencing factors included a high prevalence of macrolide-resistant epidemic strains (94.2%) and significantly higher H3N2 neutralizing antibody levels (*P* < 0.0001) stimulated by the preceding H3N2 influenza epidemic. These findings highlight the complex relationship between the prevalence of *M. pneumoniae* and H3N2 infection in children, indicating that it is necessary to consider pathogen interactions in respiratory disease management by continuously monitoring respiratory pathogens. The emergence of macrolide-resistant strains in China and the previous H3N2 influenza epidemic significantly exacerbated the severity of the *M. pneumoniae* outbreak. H3N2 infection potentially amplifies Mycoplasma transmission. This study elucidates the epidemiological and clinical aspects of *M. pneumoniae* infections in children, yields insights regarding the cause of the outbreak, and provides guidance for improving respiratory infection management.

## INTRODUCTION

*Mycoplasma pneumoniae* (*M. pneumoniae*) is a prevalent pathogen that causes respiratory infections and pneumonia in children around the world. Common symptoms of *M. pneumoniae* infection include fever, cough, and runny nose; furthermore, severe infections can lead to pneumonia and, in rare instances, death. *M. pneumoniae* infections occur year-round but often peak between August and January, with cyclical outbreaks occurring every 3–7 years (1–3).

*M. pneumoniae* is one of the smallest self-replicating organisms and has an approximately 800-kbp genome with a single copy of the P1 gene, which encodes the crucial P1 cytadhesin protein for host cell attachment. P1 integrity is vital for the attachment of virulent strains to the respiratory epithelium (4). Notably, *M. pneumoniae* strains can be classified into two major genetic groups—namely, P1 type 1 and type 2—with their prevalence varying during outbreaks. Monitoring the shift in P1 types during *M. pneumoniae* outbreaks is essential for understanding the molecular epidemiology and evolution of this pathogen. Macrolide resistance is associated with mutations in the V domain of the 23S rRNA gene, notably at positions 2063, 2064, 2067, or 2617 (5–8).

In the past 4 years, the prevalence of *M. pneumoniae* has been influenced by the emergence of SARS-CoV-2 and its associated public health interventions, such as mask-wearing mandates (9). Consequently, from April 2022 to March 2023, the incidence of *M. pneumoniae* was unexpectedly lower than that observed during epidemic periods (10–12). However, the relaxation of epidemic prevention measures has coincided with a surge in respiratory disease cases, potentially attributed to reduced human immunity following the easing of nonpharmaceutical interventions (13). Previous research has examined the phenomenon of coinfection between *M. pneumoniae* and other respiratory pathogens, and potential synergistic effects have been observed, such as increased influenza A virus titers in coinfected individuals (14). However, the interactions between successive respiratory infections have yet to be fully elucidated.

This study aims to characterize a recent outbreak of *M. pneumoniae* among children in Shanghai, China. We analyzed routine blood test results and sequence features of the *M. pneumoniae* P1 and 23S rRNA genes and assessed the correlation between *M. pneumoniae* infection and antibody titers against circulating influenza viruses and SARS-CoV-2 Omicron variants. The findings of this study should enhance our understanding of the epidemiology, clinical manifestations, and immunological responses associated with *M. pneumoniae* infections, thereby contributing to the development of more effective diagnostic and therapeutic strategies.

## MATERIALS AND METHODS

### Study design and samples

A total of 38,668 children diagnosed with respiratory tract infections were tested for *M. pneumoniae* using the simultaneous amplification and testing (SAT) kit (Shanghai Rendu Biotechnology Co., Registration No. 20173400156) at the Shanghai Children’s Medical Center in 2023. A total of 11,919 children tested positive for *M. pneumoniae* and were categorized into the *M. pneumoniae* group. To prevent subjects from experiencing co-infection with respiratory pathogens, which could lead to elevated antibody levels from ongoing infections, we conducted simultaneous tests for influenza A, influenza B, RSV (Cepheid, Registration No. 20193400413), and SARS-CoV-2 (Shanghai ZJ Bio-Tech Co., Registration No. 20203400057). Serum samples and nasopharyngeal swab samples were obtained from children (<18 years old) who visited between October and December 2023. Serum samples were inactivated at 56°C for 30 min and stored at 4°C. Nasopharyngeal swabs were stored in sample preservation solution (Shanghai Rendu Biotechnology Co., Ltd) at −80°C until further processing. Additionally, hematological results from laboratory test conducted were collected from the patient files. We assessed the severity of the patient’s illness based on clinical information about the patient recorded by the physician, such as the duration of fever days and chest imaging information.

### *M. pneumoniae* SAT

The SAT technique is a newly developed method that integrates isothermal RNA amplification with real-time fluorescence detection. In this approach, the Moloney murine leukemia virus reverse transcriptase transcribes 16S rRNA into complementary DNA (cDNA) with a T7 promoter. Subsequently, T7 RNA polymerase generates multiple RNA copies from each cDNA template. These RNA copies are then reverse transcribed into cDNA and hybridized with fluorescence-labeled specific probes, resulting in a fluorescent signal. Experimental procedures were conducted according to the manufacturer’s operating manual (Shanghai Rendu Biotechnology Co., Ltd). First, lysis buffer was added to the saline eluent from the oropharyngeal swab. The positive control consisted of *M. pneumoniae* RNA, while the negative control used saline. Next, nucleic acid extraction reagent was added to both the test sample and the controls. RNA extraction was performed using the magnetic bead method, and the RNA was eluted with detection reagents (primers, fluorescent probes, dNTPs, and NTPs). A 40 µL final detection system was prepared by mixing 30 µL of the RNA sample with 10 µL of enzyme reagent. Nucleic acid amplification was performed using a fluorescence detection instrument under the following conditions: 42°C for 40 cycles, with each cycle lasting 60 s. Results were interpreted according to the provided instructions.

### P1 gene of *M. pneumoniae* sequencing and analysis

Given the heterogeneous nature of the DNA extracted from nasopharyngeal swabs, which often contained low quantities of the target bacteria, we focused on samples that tested positive for *M. pneumoniae* via the SAT test. Specifically, we selected nasopharyngeal samples with a *M. pneumoniae*-positive result and a fluorescence channel dt value (F1 channel dt) of less than 32. This selection criterion ensured that the specimens analyzed were more likely to contain sufficient target DNA for accurate sequencing. DNA was extracted using the QIAamp DNA Mini Kit (Qiagen) following the manufacturer’s instructions. Plasmid pcDNA3.1 was utilized as the cloning vector and digested with NotI and XbaI restriction enzymes. The P1 gene of *M. pneumoniae* was amplified using PrimeSTAR Max DNA Polymerase (Takara) and cloned into the pcDNA3.1 vector using the NovoRec plus One-step PCR Cloning Kit (Novoprotein). Upstream primer P1-F (5′-AGATATCCAGCACAGTGGCGGCCGCATGCACCAAACCAAAAAAACTG-3′) and downstream primer P1-R (5′-AGCGGGTTTAAACGGGCCCTCTAGATTAGCTTCGATTTCATAAATACTAAGC-3′) were designed for P1 gene amplification. The sequence of the P1 gene was determined by TSingke Biotechnology Co. The P1 type was determined by comparing the complete P1 gene sequences using the BLAST program (https://blast.ncbi.nlm.nih.gov/Blast.cgi).

### Macrolide-resistance mutations in 23S rRNA V domain gene of *M. pneumoniae*

The 23S rRNA V domain gene was then amplified from the DNA extracted from nasopharyngeal swabs by nested PCR using the following primer sequences: MP23SV-F1 (5′-GCAGTGAAGAACGAGGGG-3′), MP23SV-R1 (5′-TCCAATAAGTCCTCGAGCAATTAGT-3′), MP23SV-B-F2 (5′-AGATATCCAGCACAGTGGCGGCCGCCCCAGTGAACGGCGG-3′), and MP23SV-B-R2 (AGCGGGTTTAAACGGGCCCTCTAGATGGGAAGATTAATCTTTGAGATAGTTTCA-3′). The first round of amplification utilized the primer pair MP23SV-F1 and MP23SV-R1. Cycling conditions involved an initial denaturation at 98°C for 5 min, followed by 50 cycles of denaturation at 98°C for 10 s, annealing at 56°C for 15 s, and extension at 72°C for 1 min. Subsequently, 8 µL of the PCR product was transferred to a second reaction using the primer combination MP23SV-B-F2/MP23SV-B-R2. The cycling protocol for the second step consisted of 40 cycles at 98°C for 10 s, 54°C for 10 s, and 72°C for 1 min. Finally, the amplified fragment was cloned into pcDNA3.1 vectors and sequenced.

### Production of influenza pseudoviruses

The gene sequences of HA and NA of influenza viruses, A(H1N1)pdm09 strain and A(H3N2) strain, were isolated from patient throat swabs collected in March 2023. The gene sequences were codon-optimized (GenScript) and integrated into the CMVR vector. Pseudoviruses for A(H1N1)pdm09 and A(H3N2) were generated by 293T cell transfection. Briefly, HEK-293T cells were co-transfected with 10 µg of HA-encoding plasmid, 10 µg of NA-encoding plasmid, 2 µg of protease-encoding plasmid, and 20 µg of pNL4-3.Luc.R-E -backbone plasmid. Cell supernatants were collected 48 hours post-transfection, and pseudoviruses were stored at −80°C. The influenza pseudoviruses were engineered to express a reporter gene, luciferase. Upon infecting host cells, the pseudovirus delivers luciferase, which integrates into the cells. The resulting cell lysate can then be analyzed using a luminometer (PerkinElmer). The luminescence generated is directly proportional to the amount of pseudovirus present, enabling a rapid and sensitive quantitative assessment of neutralizing antibody titers. The titers of sera were calculated as ID50 and expressed as the highest dilution of plasma, which results in a 50% reduction of luciferase luminescence compared with virus control (15).

### Influenza virus neutralization assay

The neutralizing activities of serum samples against influenza A(H1N1)pdm09 and A(H3N2) were assessed using single-round pseudovirus infection of 293T cells as previously reported (15). Initially, 2 × 10^5^ HEK-293T cells were seeded in a 96-well plate and incubated at 37°C for 16 hours. Serum samples were diluted 1:100 and then subjected to fourfold serial dilutions. The medium in the 293T cells was aspirated, and 50 µL of the diluted serum samples was added to the designated wells along with 50 µL of pseudoviruses. Additional fresh medium was added 24 hours later. At 48 hours post-infection, the cells were lysed with 50 µL lysis buffer. A volume of 30 µL of the lysates was transferred to 96-well flat-bottom plates (Costar) to detecte the relative light units using a firefly luciferase assay kit (Promega) on PerkinElmer Ensight. When neutralizing antibodies (nAbs) bind to the pseudovirus, nAbs inhibit the ability of pseudoviruses to infect target cells, thereby reducing luciferase expression, which reflects the level of successful infection. Luciferase activity correlates inversely with neutralizing-antibody titer in assays utilizing pseudoviruses. As the titer of neutralizing antibodies increases, luciferase activity decreases, indicating effective neutralization. A nonlinear regression analysis was performed on the resulting curves, using Prism 9.5.0 (GraphPad), to calculate the median 50% inhibitory dilution (ID50).

### Production of SARS-CoV-2 pseudoviruses

The Spike genes of BA.5, EG.5, and XBB.1.16 were cloned in the pcDNA3.1 vector. Then, 4.5 µg plasmids expressing spike protein of SARS-CoV-2 and 18 µg pNL4-3.Luc.R-E-backbone plasmid were transiently co-transfected into 293T cells using EZ *Trans* transfection reagent (Life-iLab). After 48 hours, the supernatants were harvested and stored at −80°C until use.

### SARS-CoV-2 neutralization assay

The neutralization activities of sera against SARS-CoV-2 were determined using Huh-7 cells as previously described (16). The serum samples were initially diluted to 1:100 and then subjected to fourfold serial dilutions. Subsequently, 10 µL of the diluted sera was mixed with 40 µL of pseudovirus in a 96-well plate and incubated at 37°C for 30 min. Following this incubation, 1 × 10^4^ Huh-7 cells were added to the mixture and cultured for 48 hours in a 37°C incubator. After 24 hours of infection, the cells were supplemented with 150 µL of fresh growth medium. Following another 48 hours of incubation, the supernatants were removed, and the Huh-7 cells were lysed to evaluate luciferase expression. ID50 was calculated as the serum dilution that reduced infection by 50% in Prism 9.5.0 (GraphPad).

### Statistical analysis

Data collection was conducted using Excel 2019, while data analyses were performed using GraphPad Prism 9.5.0 (GraphPad Software Inc, San Diego, CA, USA). Seropositivity was defined as a neutralization antibody titer of 40 or higher. Due to the non-normal distribution of antibody titers, single comparisons between two independent groups were assessed using the Mann-Whitney *U* test. A *P* value of < 0.05 was considered statistically significant. Descriptive analyses of participant characteristics and blood routine data were presented using medians (IQR) for continuous variables and counts (%) for categorical variables.

## RESULTS

### The epidemiology of *M. pneumoniae* infection among children in China

**Fig 1:**
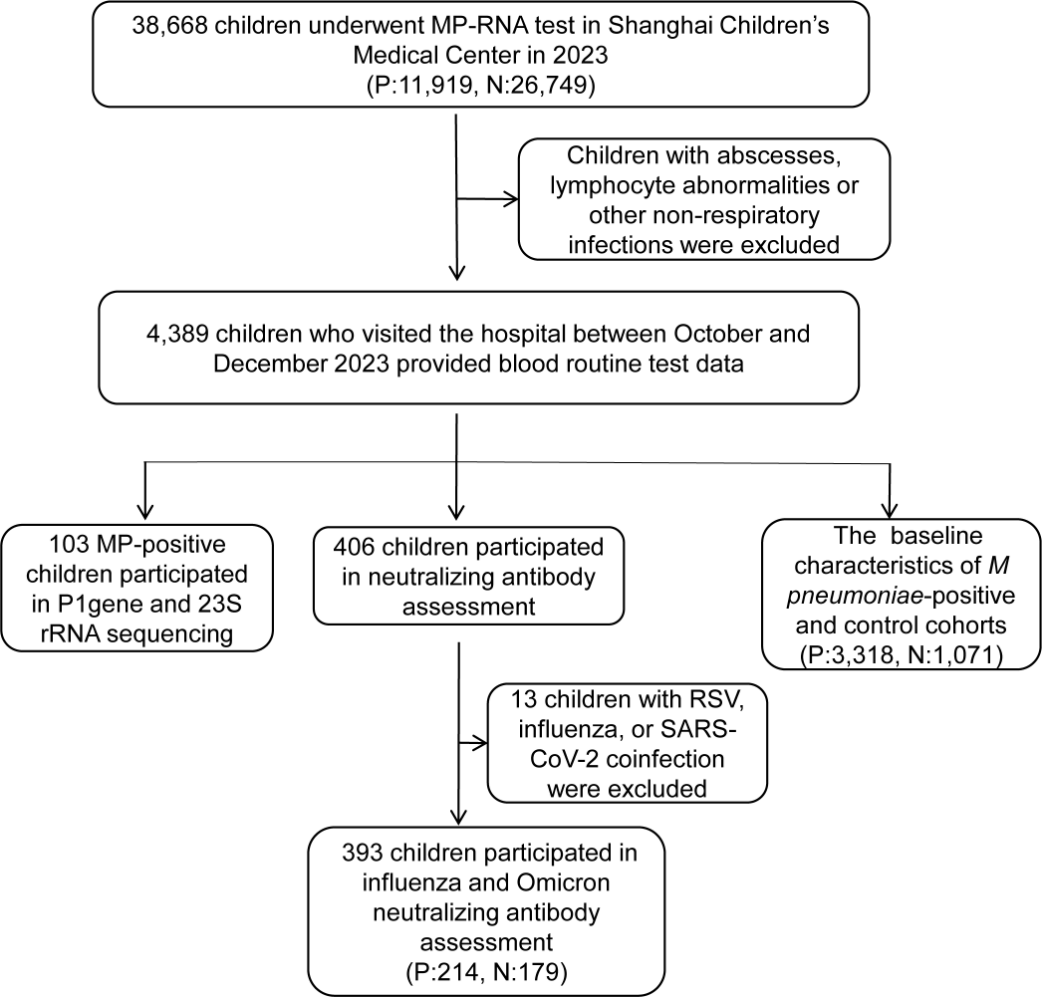
Flowchart of the study. In 2023, 38,668 PCR tests at Shanghai Children’s Medical Center revealed 11,919 *M*. *pneumoniae*-positive cases. One hundred three swab samples underwent gene sequencing, and serum samples from 214 positive and 179 negative children were tested for neutralizing activity against influenza and Omicron variants. Clinical data from 3,318 positive and 1,071 negative children were compared. MP, *Mycoplasma pneumoniae*.

**Fig 2:**
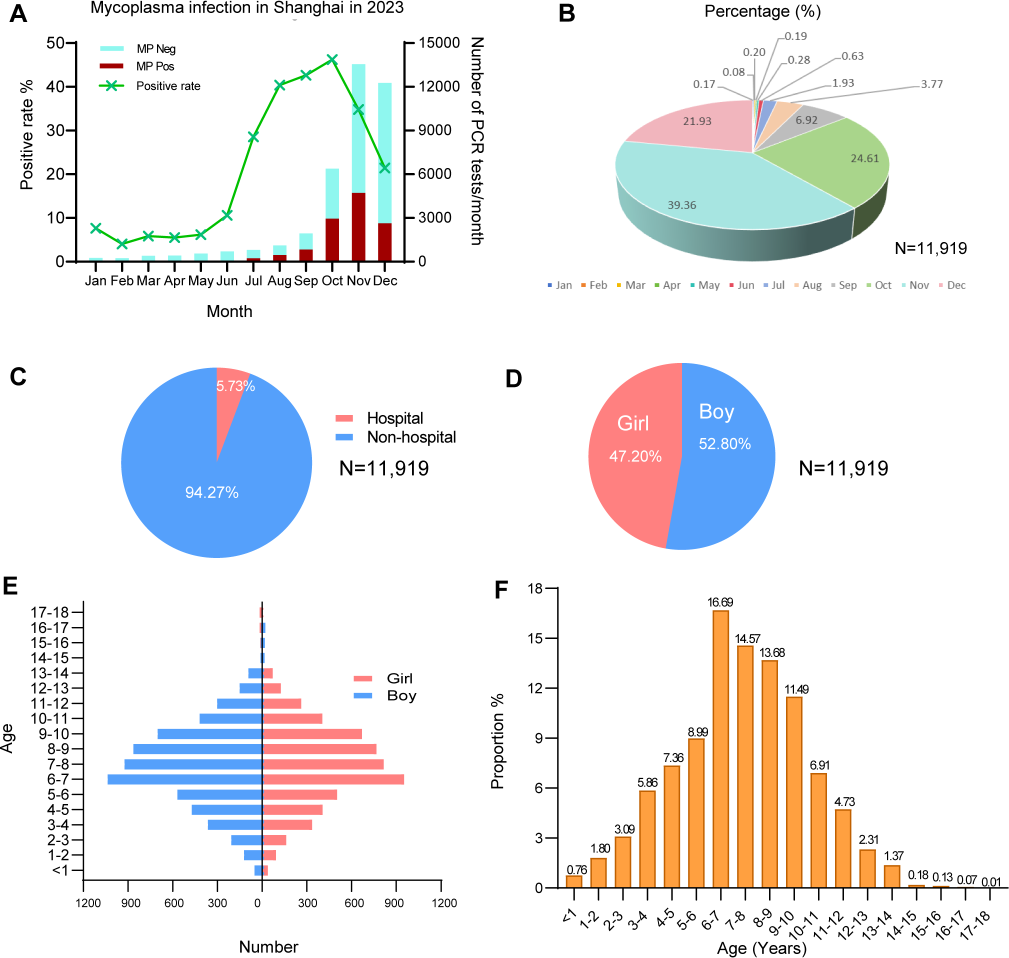
The epidemiology of *M. pneumoniae* in children in 2023. (**A**) The monthly number of PCR tests and the positive rate for *M. pneumoniae* infection in Shanghai, China during 2023. (**B**) The distribution and proportion of *M. pneumoniae*-positive children in different months. (**C**) The proportion of hospitalized children among *M. pneumoniae*-positive cases. (**D**) The proportion and (**E**) distribution of gender among *M. pneumoniae*-positive children. (**F**) The positive rate of *M. pneumoniae* infection among children at different ages.

In 2023, 38,668 PCR tests were conducted at the Shanghai Children’s Medical Center, and 11,919 (30.8%) children tested positive for *M. pneumoniae*. Owing to the remarkably high *M. pneumoniae* positivity rate, which exceeded 40% of the tested population in August and September 2023 (Fig. 2A), we initiated sample collection and research promptly after observing this phenomenon, with the aim of elucidating the possible causes of this outbreak. Clinical characteristics were analyzed by comparing 3,318 children with *M. pneumoniae* infection and 1,071 *M*. *pneumoniae*-negative children who visited the clinic between October and December 2023 and underwent blood tests. A total of 103 positive swab samples were subjected to P1 and 23S rRNA gene sequencing. Serum samples from 214 *M*. *pneumoniae*-positive and 179 *M*. *pneumoniae*-negative children were tested for neutralizing activity against circulating influenza A (H1N1)pdm09, A(H3N2), and the SARS-CoV-2 Omicron variants BA.5, XBB.1.16, and EG.5. The flowchart of the study procedure is presented in Fig. 1.

A significant increase in the detection rate of *M. pneumoniae* was observed from July 2023 onward, with the percentage of positive cases reaching 46.2%. This finding is in contrast to the period between January and June 2023, when the positive rate ranged from 5% to 10% (Fig. 2A). The median age of the 11,919 *M*. *pneumoniae*-positive children was 7.33 years (IQR, 5.67–9.17 years). A total of 86.0% of the *M. pneumoniae*-positive children were infected between October and December 2023 (Fig. 2A and B), and 5.73% of them were hospitalized (Fig. 2C). Among the *M. pneumoniae*-positive children, 52.8% were boys (Fig. 2D), and the distribution of sex across different age groups was similar (Fig. 2E). Additionally, more than 65% of the *M. pneumoniae*-positive children were between 5 and 10 years old (Fig. 2F).

### P1 gene sequencing and phylogenetic analysis

**Fig 3:**
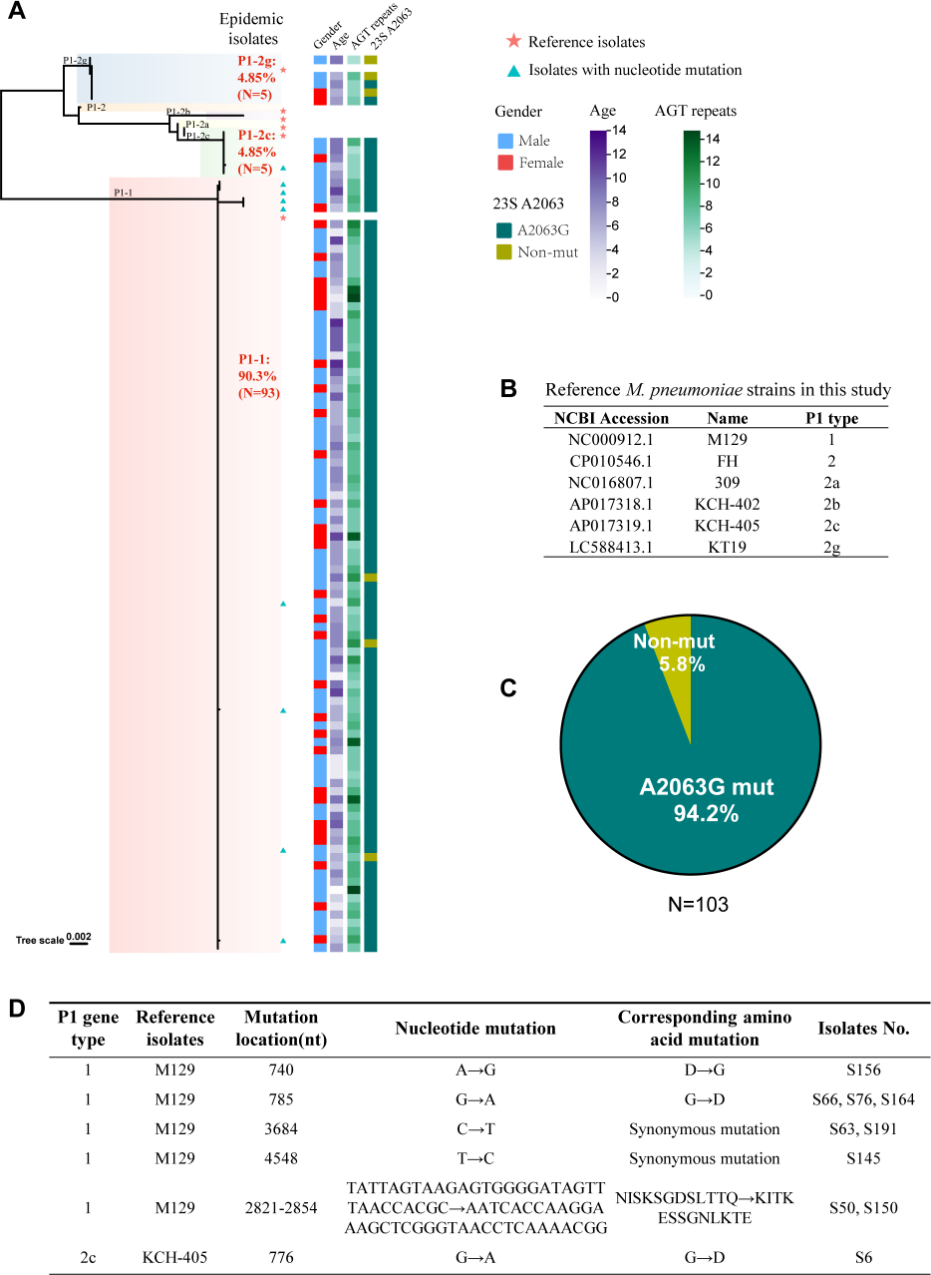
Phylogeny of *M. pneumoniae* in children. (**A**) Phylogenetic tree of *M. pneumoniae* Isolates generated by MEGA software. Isolates of the same type were labeled with the same color. The strains marked with red stars are the reference strains. The strains marked with green triangles are those in which nucleotide mutations were found. (**B**) Reference *M. pneumoniae* strains included in this study. (**C**) The pie chart represents the proportion of mutations at the 23S rRNA gene A2063 site in 103 *M*. *pneumoniae* isolates. (**D**) Nucleotide mutations and corresponding amino acid changes in the P1 genes of *M. pneumoniae* strains inconsistent with the strains reported.

To comprehensively assess the epidemic status of *M. pneumoniae*, we conducted a detailed analysis of the entire P1 gene sequence from 103 children who tested positive for *M. pneumoniae* RNA via PCR and presented with respiratory symptoms (Fig. 3A; Table S1). Among these children, 69 were boys and 34 were girls, and their ages ranged from 0 to 12 years (Fig. 3A; Table S1). The amplified sequences were compared with *M. pneumoniae* genomes from NCBI databases (Fig. 3B), and reference strains were categorized into distinct subtypes based on established classifications (Fig. 3B) (17–21).

The P1 isolates can be categorized into two groups—namely, type 1 and type 2—along with their respective variants, on the basis of PCR-RFLP divergence results of the P1 sequence. Among the isolates, 93 (90.29%) were identified as type 1 (Fig. 3A). The remaining isolates belonged to type 2, including five (4.85%) type 2c isolates and five (4.85%) type 2g isolates (Fig. 3A). These results indicate that type 1 strains of *M. pneumoniae* were predominant in Shanghai in 2023.

Another significant feature within the P1 gene is the AGT trinucleotide variable number tandem repeat, which encodes serine in *M. pneumoniae*. The number of these repeats varied from 5 to 15 among the isolates in our study (Fig. 3A), with no discernible differences observed in patients on the basis of the varying repeat numbers.

Furthermore, we identified 10 isolates with mutations that did not precisely match any previously reported sequences (Fig. 3D). For example, in isolate S156, a nucleotide mutation was detected at position 740 nt (G→A), resulting in a corresponding amino acid mutation from aspartic acid to glycine. Similarly, a synonymous point mutation was identified in isolate S145 at nucleotide position 4548 nt (T→C). These findings underscore the importance of monitoring potential mutations within the previously conserved P1 protein, as these mutations may contribute to the emergence and spread of *M. pneumoniae*.

### Macrolide resistance mutations in the 23S rRNA V domain gene of *M. pneumoniae*

A mutation in the V domain of 23S rRNA is responsible for conferring phenotypic resistance to macrolides. Next, we assessed the prevalence of macrolide resistance in circulating *M. pneumoniae* strains in China. Among the 103 *M*. *pneumoniae-*positive samples analyzed herein, 97 (94.2%) harbored the A2063G mutation in the V domain of the 23S rRNA gene (Fig. 3A and C; Table S1). Furthermore, with the exception of the prevalent A2063G mutation, no base substitutions were detected at other known hotspot mutation sites. This observation is consistent with the predominance of the A2063G mutation, thus underscoring the importance of this mutation in mediating macrolide-resistant *M. pneumoniae* (MRMP), whereas mutations at other sites are comparatively less frequent. However, importantly, the presence of drug-resistant *M. pneumoniae* is not always correlated with clinical outcomes. Some patients infected with drug-resistant strains showed clinical improvement after macrolide treatment, including resolution of fever and alleviation of cough, particularly with azithromycin. In contrast, other patients continued to experience fever and persistent cough despite macrolide treatment and were subsequently treated with minocycline (Table S1). This variability underscores the complexity of clinical responses to drug-resistant *M. pneumoniae* infections.

### Enhanced neutralizing antibody response to the H3N2 influenza strain in *M. pneumoniae*-positive patients

**Fig 4:**
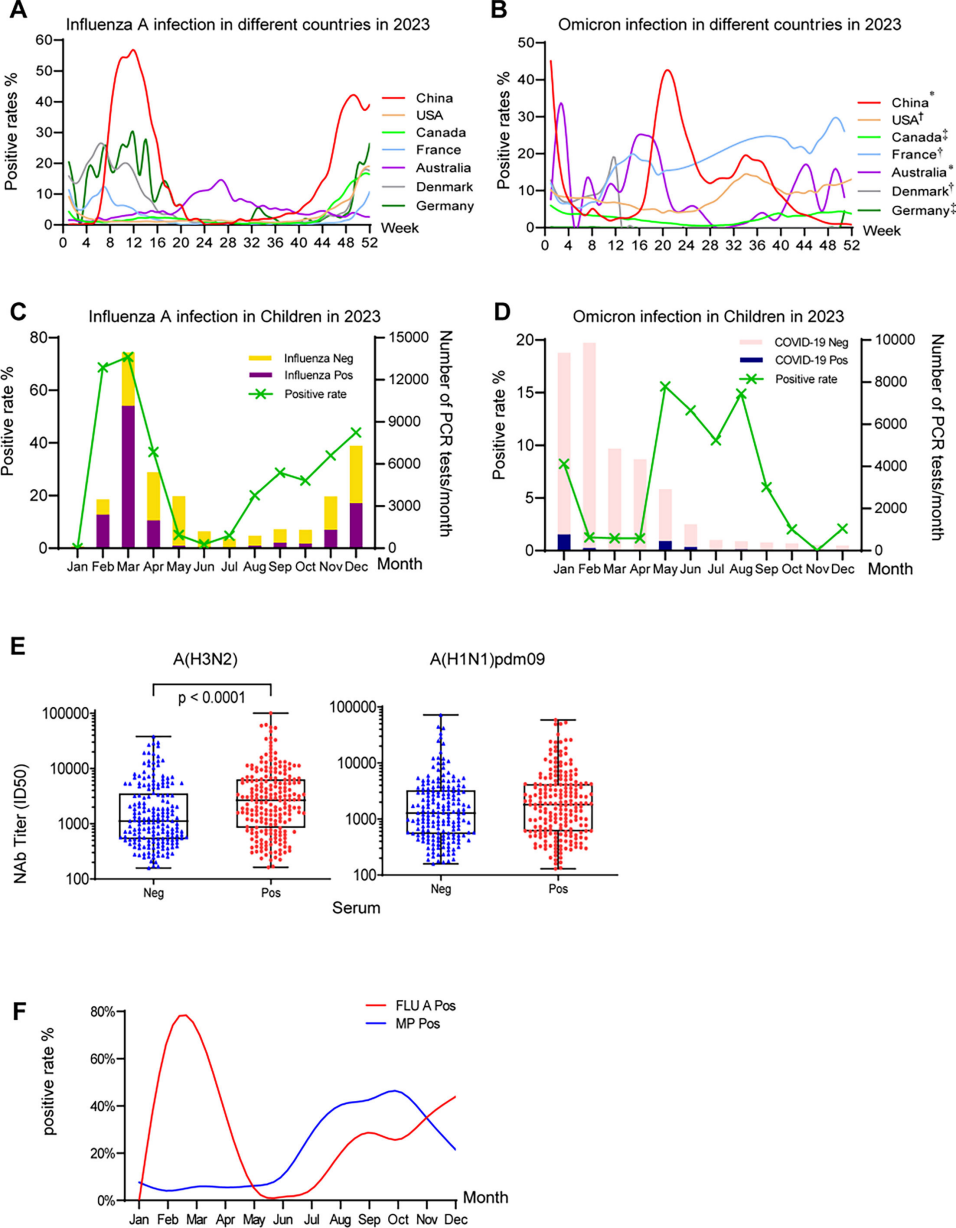
Neutralization antibody titers of patient serum against circulating influenza viruses and Omicron variants in children. Positive rates of (**A**) influenza A virus infection (22) and (**B**) Omicron infection in China and different countries during 2023 (23–28). The data marked with * for China and Australia represent the positivity rate of COVID-19 among flu-like patients, while † for the United States, France, and Denmark represents the laboratory-tested positivity rate of COVID-19. The data marked ‡ for Germany and Canada represent the overall population positivity rate of COVID-19. The number of PCR tests per month and positive rate for (**C**) influenza A virus infection, and (**D**) Omicron infection in Shanghai Children’s Medical Center, China during 2023. The major circulating variants are labeled on the top of the peaks. (**E**) Neutralizing antibody titers of patient sera against the circulating A(H3N2) and A(H1N1)pdm09 strains. A total of 214 serum samples from *M. pneumoniae*-positive patients and 179 control serum samples were included. *P*-values were calculated using *t*-tests. (**F**) Comparison of positive rates for influenza A infection and *M. pneumoniae* infection in 2023.

The outbreak of *M. pneumoniae* infection followed previous waves of influenza A and SARS-CoV-2 Omicron outbreaks in China. We analyzed the positivity rates of influenza A and SARS-CoV-2 Omicron across different countries and found that China exhibited the highest rates. The positivity rate of influenza A reached 55.85% in China (Fig. 4A), whereas the positivity rate of SARS-CoV-2 Omicron was 45% (Fig. 4B). Similarly, in Shanghai, a significant resurgence of influenza A (H1N1)pdm09 and influenza A (H3N2) coinfections occurred from February to May 2023 in children, with a positive rate of up to 72.7% (Fig. 4C). A second wave of H3N2 infection was observed from August to December 2023, with a positivity rate of up to 40%. Following the easing of the epidemic situation in December 2022, an epidemic of SARS-CoV-2 Omicron variants BA.5, XBB.1.16, and EG.5 occurred in Shanghai in 2023. Three waves of SARS-CoV-2 Omicron infections were observed in January, May, and August 2023 (Fig. 4D). To explore the complex relationships among influenza A, SARS-CoV-2 Omicron variants, and *M. pneumoniae* infections, we collected serum samples from 214 *M*. *pneumoniae*-positive children and 179 *M*. *pneumoniae*-negative children to assess their neutralization antibody titers against preceding influenza strains A(H1N1)pdm09 and A(H3N2), as well as SARS-CoV-2 Omicron subvariants in 2023 in Shanghai. All participants tested negative for influenza A, influenza B, and SARS-CoV-2 via PCR on the day of sample collection (Table S2). Compared with control patients, patients with *M. pneumoniae* infection presented significantly higher neutralizing antibody levels against H3N2 in Shanghai in 2023 (*P* < 0.0001, Fig. 4E), whereas no difference was observed for H1N1pdm09 (Fig. 4E) or SARS-CoV-2 Omicron BA.5, XBB.1.16, and EG.5 variants (Table S2). These findings indicate a potential link between prior influenza A (H3N2) infection and subsequent *M. pneumoniae* infection. H3N2 infection may facilitate the development of *M. pneumoniae* infection. Furthermore, when we overlaid the prevalence curves of *M. pneumoniae* and H3N2 in children, a parallel trend was observed from June to December 2023 (Fig. 4F), suggesting a potential association between *M. pneumoniae* infection and H3N2 infection. This observation suggests that outbreaks of *M. pneumoniae* may be related not only to the prevalence of resistant strains but also to potential viral–bacterial interactions between influenza A (H3N2) infection and *M. pneumoniae* infection.

### Clinical characteristics of *M. pneumoniae*-positive patients

To evaluate the synergistic impact of macrolide resistance and H3N2 infection on the outcomes of *M. pneumoniae* infection, we compared the clinical manifestations of 3,318 *M*. *pneumoniae*-positive and 1,071 *M*. *pneumoniae*-negative children who visited the hospital between October and December 2023 and underwent routine blood tests. The demographic and baseline clinical characteristics are presented in Table 1. There was no significant difference between the *M. pneumoniae*-positive group and the control group in terms of age (7.3 [IQR, 5. 7–9.2] vs 7.3 [IQR, 5.9–9.5], *P* = 0.27), sex distribution (male 1,723 [51.9%] vs male 541 [50.5%], *P* = 0.44), and hospitalization rate (306 [9.2%] vs 114 [10.6%], *P* = 0.17). These demographic findings provide a comprehensive profile of the study group, thus facilitating a nuanced analysis of *M. pneumoniae* infection dynamics in the pediatric cohort. Compared with the control group, the *M. pneumoniae*-positive group presented significantly elevated CRP levels, neutrophil counts, and monocyte counts (all *P* < 0.0001, Table 1). Conversely, there was a notable decrease in lymphocytes, basophils, and eosinophils in *M. pneumoniae*-positive children (all *P* < 0.0001, Table 1). These data suggest that infection with MRMP in children who have been previously infected with influenza A (H3N2) stimulates a significantly strong inflammatory response, induces considerable damage to the immune system, and amplifies Mycoplasma transmission. These findings highlight the importance of considering broader immunological consequences when evaluating the prevalence of *M. pneumoniae* infection among affected children.

**TABLE 1:** Baseline characteristics of the *M. pneumonia-*positive and control cohorts^*a*^

Parameters	Positive (*n* = 3,319)	Negative (*n* = 1,071)	*P* value
Age (years); median (IQR)	7.3 (5.7–9.2)	7.3 (5.9–9.5)	0.27
Boys	1,723 (51.9%)	541 (50.5%)	0.44
Girls	1,596 (48.1%)	530 (49.5%)	
Hospital	306 (9.2%)	114 (10.6%)	0.17
Laboratory markers (median [IQR])			
CRP (mg/L)	7.6 (3.2–15.0)	3.2 (0.8–8.9)	<0.0001
Neutrophil (%)	59.6 (51.1–66.6)	56.0 (43.6–66.0)	<0.0001
Monocyte (%)	8.6 (6.9–10.5)	8.0 (6.3–10.6)	0.0001
Lymphocyte (%)	29.1 (22.9–37.0)	32.3 (22.7–43.4)	<0.0001
Basophil (%)	0.2 (0.1–0.3)	0.3 (0.2–0.4)	<0.0001
Eosinophil (%)	1.1 (0.4–2.4)	1.7 (0.6–3.6)	<0.0001
RDW (fL)	12.8 (12.3–13.3)	12.9 (12.5–13.4)	<0.0001
MCHC (g/dL)	327 (322–333)	329 (323–334)	<0.0001
WBC count (×10^9^/L)	7.9 (6.4–9.8)	8.1 (6.1–10.5)	0.50
RBC count (×10^12^/L)	4.7 (4.5–5.0)	4.7 (4.5–5.0)	0.14
PDW (fL)	10.4 (9.5–11.6)	10.5 (9.5–11.6)	0.30
Hematocrit (%)	39.9 (38.1–41.8)	40.1 (38.2–41.8)	0.33
Hemoglobin (g/L)	131 (125–138)	131 (126–138)	0.01
MCH (pg)	27.8 (27.0–28.6)	27.9 (27.1–28.7)	0.18
MCV (fL)	84.8 (82.3–87.3)	84.5 (82.2–87.0)	0.14
MPV (fL)	9.7 (9.2–10.4)	9.8 (9.2–10.4)	0.55
Platelets (×10^9^/L)	255 (208–320)	264 (214–324)	0.04

^*a*^ PDW, platelet distribution width; RBC, red blood cell; WBC, white blood cell count; MCH, mean corpuscular hemoglobin; MCHC, mean corpuscular hemoglobin concentration; MCV, mean corpuscular volume; MPV, mean platelet volume; and RDW, red blood cell volume distribution width.

## DISCUSSION

*M. pneumoniae* is a common pathogen that causes respiratory infections in children (10). During the COVID-19 pandemic, the incidence of *M. pneumoniae* decreased significantly following the implementation of nonpharmaceutical interventions (11). However, the outbreak of *M. pneumoniae* resurfaced after the pandemic, which researchers attributed to the reduced human immunity after the easing of nonpharmaceutical interventions and the spread of MRMP (3, 29–31). We aimed to explore whether coinfection with other respiratory pathogens influences the prevalence and severity of *M. pneumoniae* infections. Therefore, we conducted a comprehensive series of analyses, including routine blood tests, genomic sequencing of the *M. pneumoniae* P1 gene, and measurements of neutralizing antibody levels against SARS-CoV-2 Omicron variants and influenza A.

Our study included 11,919 *M*. *pneumoniae*-positive children and revealed that *M. pneumoniae* was more frequently detected in children aged 5–10 years. There were no sex-based differences in detection rates. These findings highlight the notable demographic differences in *M. pneumoniae* infections during this period. Furthermore, analysis of routine blood data from 4,389 children revealed distinctive immunological responses in *M. pneumoniae*-positive individuals. These patients presented significantly elevated CRP and neutrophil levels, indicating an inflammatory response consistent with the acute phase of infection (32). Conversely, there was a significant reduction in lymphocyte, basophil, and eosinophil counts, which suggested potential immune dysregulation due to the interference of the pathogen with immune cell function or recruitment. These findings emphasize the complex interaction between *M. pneumoniae* and the host immune system. Longitudinal studies tracking changes in blood parameters over the course of *M. pneumoniae* infection may provide insights into the progression and resolution of the inflammatory response. However, although there were differences in certain routine blood examination indicators between the *M. pneumoniae*-positive group and the *M. pneumoniae*-negative group, the routine blood parameters for children in the *M. pneumoniae*-positive group and the *M. pneumoniae*-negative group remained within the normal reference ranges. These findings suggest that during the epidemic season of *M. pneumoniae*, it is essential to conduct targeted tests for this virus in children whose routine blood parameters are within the normal reference ranges. Additionally, there should be an active search for the possibility of other pathogen infections or coinfections.

We analyzed 103 *M*. *pneumoniae* RNA-positive samples and reported that the predominant subtype in Shanghai was type 1, consistent with previous findings from Suzhou, China, in late 2023 (29). It has been proposed that the cyclical epidemics of *M. pneumoniae*, which typically occur every few years, may be linked to a shift between P1 subtypes (33). These two primary subtypes are immunologically distinct, and exposure to one may induce temporary herd immunity, thereby suppressing infections of that subtype while allowing the other to reemerge (34). However, there have also been reports suggesting the possibility of the simultaneous occurrence of both subtypes (35). In the cohort involved in this study, nearly half (47/93, 50.5%) of the patients with the P1-1 subtype had pneumonia; similarly, 4 out of 10 patients with the P1-2 subtype developed pneumonia (Table S1), suggesting that the P1 type may not be directly correlated with pathogenicity (36). However, owing to the relatively small number of people in the P1-2 group, the differences between type 1 and type 2 were not examined in this paper and thus have yet to be elucidated. Subtype shifts and the emergence of new mutations in the P1 protein raise concerns about antigenicity and the possibility of future epidemics (33). Therefore, ongoing monitoring and characterization of these mutations are crucial for understanding their implications for disease transmission and severity.

In terms of drug resistance, more than 94.2% (97/103) of the *M. pneumoniae* strains in this study were resistant to macrolides. Notably, macrolide resistance rates vary among different countries, with China exhibiting an exceptionally high rate (37). Similar to the high macrolide resistance rates reported in this study, Asian countries, including China, Japan, and South Korea, have reported high rates of macrolide resistance (38–41). However, macrolide resistance rates for *M. pneumoniae* have been reported to be low in the USA (6), Spain (42), and Europe (43). The persistence of MRMP isolates in China highlights the importance of ongoing surveillance, coupled with the development of rapid diagnostic tools and research efforts to combat antimicrobial resistance in *M. pneumoniae* infections. A coordinated surveillance program is essential for curbing the spread of antibiotic resistance in countries with low macrolide resistance rates for *M. pneumoniae*. Notably, when macrolide resistance was targeted, the drug resistance of *M. pneumoniae* was not entirely consistent with clinical outcomes. Some resistant patients exhibited normal body temperature recovery and the resolution of lung rales after the use of macrolide antibiotics (Table S1). However, there are also patients in clinical practice whose conditions did not improve after the use of macrolide antibiotics (Table S2). Therefore, minocycline is used as an alternative to macrolide antibiotics for treatment. Although minocycline may lead to tooth discoloration or enamel hypoplasia, owing to resistance to macrolide antibiotics and for the purpose of effectively controlling the disease and preventing its further deterioration, the use of minocycline should be cautiously considered in clinical practice.

Influenza virus is one of the leading causes of severe community-acquired pneumonia in China, and its prevalence rate varies across seasons (44). Studies have shown that seasonal influenza poses a greater risk of pulmonary complications, such as cough, hypoxia, and shortness of breath. In contrast, COVID-19 has a greater risk of mortality and multiorgan involvement, excluding the pulmonary system (45). Our research indicated that children infected with *M. pneumoniae* had notably elevated neutralizing antibody titers against H3N2 (*P* < 0.0001), which was related to the prevalence of influenza A from January to May 2023. These findings suggest a potential correlation between H3N2 and *M. pneumoniae* infections. Specifically, H3N2 infections might compromise the pulmonary system, thereby increasing the susceptibility of children to *M. pneumoniae*. First, H3N2 infection can induce inflammation and damage respiratory epithelial cells, thus creating a more conducive environment for the growth of Mycoplasma. Second, H3N2 infection might influence the host’s defense against Mycoplasma by modulating the immune response and destroying the immune system, thus increasing the susceptibility of individuals to Mycoplasma infection and transmission. Parallelism between the prevalence curves of *M. pneumoniae* and H3N2 in 2023 (Fig. 4F) adds an intriguing aspect to our study, suggesting potential interactions between these two pathogens. This parallel trend raises questions about the underlying mechanisms driving their co-occurrence and whether there might be shared environmental or host factors influencing their transmission dynamics. Further investigations into these factors could provide valuable insights into the epidemiology of respiratory infections and aid in the development of targeted intervention strategies. Additionally, meteorological variables, such as temperature, humidity, and decreased air quality, may play a significant role in increasing susceptibility to respiratory pathogens (46, 47). Animal studies have indicated that sex, age, genetic background, and environmental stress can be risk factors for *M. pneumoniae* infection. These elements may help explain the diverse clinical manifestations observed in human disease (48).

Our study has the following limitations. First, we did not investigate the specific mechanisms by which H3N2 infection affects *M. pneumoniae*. Understanding these interactions is vital for developing effective treatment strategies. Second, our research was conducted at a single center focused on a pediatric cohort in Shanghai, China, which may limit the generalizability of our findings. Future studies should include diverse geographic areas and populations to better assess the global characteristics of *M. pneumoniae* infection. Finally, long-term follow-up studies are needed to explore the sustained effects of *M. pneumoniae* infection on host development, providing valuable insights into the potential long-term effects of *M. pneumoniae* infection on respiratory health in affected individuals.

In summary, the emergence of macrolide-resistant epidemic strains in China, coupled with the earlier H3N2 influenza epidemic, significantly contributed to Mycoplasma transmission and *M. pneumoniae* outbreaks in China. Our findings emphasized the complex relationship between the prevalence of *M. pneumoniae* and the prevalence of H3N2 infections, thus highlighting the need to consider these interactions in the management of respiratory diseases. Therefore, continuous surveillance of respiratory pathogens is crucial. Ongoing research in this field will not only enhance our understanding of respiratory virus dynamics but also guide the development of more targeted and effective public health interventions.

## Data Availability

The sequencing data reported in this study have been deposited in GenBank under the following accession numbers: PV037255–PV037357 (P1 gene of *M. pneumoniae*) and PV017903–PV018005 (23S rRNA V domain gene of *M. pneumoniae*). All other data supporting the findings of this study are included in the article and are available from the corresponding author upon request.

## SUPPLEMENTAL MATERIAL

Supplemental material spectrum.01844-24-s0001.docx Tables S1 and S2; Figure S1.

ASM does not own the copyrights to Supplemental Material that may be linked to, or accessed through, an article. The authors have granted ASM a non-exclusive, world-wide license to publish the Supplemental Material files. Please contact the corresponding author directly for reuse.
