# Supplementary material for: Synergistic impact of macrolide resistance and H3N2 infection on M. pneumoniae outbreak in children
Source: Microbiol Spectr. 2025 Feb 25;13(4):e01844-24. doi: 10.1128/spectrum.01844-24 (PMC11960130; doi:10.1128/spectrum.01844-24)
Supplement: Supplemental material — Tables S1 and S2; Figure S1. [file spectrum.01844-24-s0001.docx]

Supplementary Table 1. *M. pneumoniae* P1 subtyping and MRMP mutations in 103 positive children

Supplementary Table 2. Neutralizing antibody responses against Influenza A and Omicron variants in *M. pneumoniae* positive and negative children

Supplementary Figure 1. Neutralizing antibody titers of patient sera against the the Omicron variants BA.5, XBB.1.16, and EG.5. The study included 214 serum samples from *M. pneumoniae*-positive patients and 179 control serum samples. *p*-values were calculated using t-tests.
